# Safety and efficacy of Chinese herbal tonics for the treatment of olfactory disorders caused by the Delta strain in COVID-19: A protocol for systematic review and meta-analysis

**DOI:** 10.1097/MD.0000000000031827

**Published:** 2022-12-02

**Authors:** Qing Gao, YiFeng Wang, Xuhao Li

**Affiliations:** a College of Acupuncture and Tuina, Shandong University of Traditional Chinese Medicine, Jinan, Shandong, China.

**Keywords:** Chinese herbal tonics, coronavirus disease 2019, olfactory disorders, protocol

## Abstract

Coronavirus disease 2019 (COVID-19) is an acute respiratory infectious disease that makes breathing difficult and is often accompanied by olfactory disorders and distension. Chinese herbal tonics, a special external treatment of traditional Chinese medicine, has shown beneficial effects in the treatment of olfactory disorders. Currently, there is a lack of systematic reviews on Chinese herbal tonics for the treatment of olfactory disorders. We conduct this study to evaluate the efficacy and safety of Chinese herbal tonics in the treatment of olfactory disorders. This study is designed to evaluate the effectiveness and safety of Chinese herbal tonics for olfactory disorders in COVID-19.

**Methods:**

Randomized controlled trials from December 2019 to September 2022. will be included, without restrictions on language or publication date. PubMed, EMBASE, Cochrane Library, Web of Science, Chinese Biomedical Databases, China National Knowledge Infrastructure, Wanfang Database, and VIP Database were searched. Two researchers will independently select studies, extract data, and evaluate study quality. The Cochrane risk of bias tool for randomized trials will be used to assess the risk of bias in the included studies. Statistical analyses will be conducted using the Review Manager (RevMan 5.3, Cochrane Collaboration, Nordic Cochrane Center, Copenhagen, Denmark).

**Results:**

This study aimed to prove the efficacy and safety of Chinese herbal tonics for olfactory disorders in patients with COVID-19. Our study provides a more accurate treatment method for olfactory disorders during COVID-19.We will publish our results in a peer-reviewed journal.

## 1. Introduction

Coronavirus disease 2019 (COVID-19) is caused by severe acute respiratory syndrome coronavirus 2 (SARS-CoV-2) infection with high infectivity and tall mortality, and it has rapidly developed into a worldwide public health emergencies in the world. Multiple studies have reported olfactory disorders symptoms in COVID-19.^[1]^ It was only later that an association between COVID-19 and olfactory and gustatory disturbances during and after coronavirus infection was described. Loss of smell is considered a symptom of COVID-19 based on a growing body of evidence, including a meta-analysis showing that 55% (95% confidence interval 38%–70%) of patients with COVID-19 have loss of smell.^[2,3]^

The report noted that olfactory disorders are one of the common symptoms of post-acute sequelae of SARS-CoV-2 infection.^[4]^ According to a recent report, olfactory disorders are a common symptom in delta-variant cases.^[5]^ Currently, the treatment methods for olfactory disorders in COVID-19 are mainly Western medicines, which may prolong viral replication in SARS-CoV2 and might be associated with a worse COVID-19 clinical course.^[6]^ Therefore, alleviating olfactory disorders in COVID-19 patients helps improve the quality of life.^[7]^

In recent years, researchers have conducted a large number of clinical trials of herbal tonics for the treatment of viral infectious diseases, mainly focusing on the treatment of influenza, new crown pneumonia, and hand, foot, and mouth disease, and more Meta-analyses/systematic evaluations evaluating the efficacy of herbal tonics have been published, but these Meta-analyses/systematic evaluations differ in the original literature included, study methods, and outcome indicators, and the conclusions obtained However, these meta-analyses/systematic evaluations differed in terms of the original literature, study methods, outcome indicators, and the conclusions obtained.^[8]^

As one of the external treatment methods of Traditional Chinese medicine, Chinese herbal tonics has the functions of dredging meridians, promoting qi and blood circulation, channeling meridians, activating collaterals and relieving pai. Studies have shown that Chinese herbal tonics has unique advantages in the treatment of olfactory disorders and is widely used worldwide.^[8]^ Stimulating acupoints can release endogenous opioid polypeptides from periaqueductal gray matter and activate opioid receptors in the central nervous system to produce labor pain by simulating the endogenous antipain substance enkephalin. During the COVID-19 epidemic, Chinese herbal tonics has been used as a complementary treatment for COVID-19 in China and has been confirmed the efficacy of COVID-19 with routine regimens. According to published studies, Chinese herbal tonics can effectively relieve the symptoms of olfactory disorders in COVID-19, reduce the frequency of olfactory disorders, and shorten the duration of olfactory disorders;^[9]^ however, high-quality evidence is lacking to support the effectiveness and safety of Chinese herbal tonics for olfactory disorders. Therefore, we designed this study to better understand the effectiveness and safety of Chinese herbal tonics therapy for olfactory disorders in COVID-19.

In this paper, we analyzed the methodological and outcome indicator evidence quality of currently published clinical randomized controlled trials of herbal tonics for the treatment of COVID-19, with the aim of providing evidence-based evidence for the clinical application of herbal tonics.

## 2. Methods

### 2.1. Study registration

This systematic review protocol was registered with PROSPERO (No. CRD42022368715). We will follow the recommendations outlined in the Cochrane Handbook of Systematic Review of Interventions and the preferred reporting items for systematic reviews and meta-analysis protocols (PRISMA-P) statement guidelines. If amendments are required, we will update our protocol to include any changes in the entire research process.

### 2.2. Criteria for including studies

#### 2.2.1. Types of studies.

Randomized controlled trials of Chinese herbal tonics in the treatment of olfactory disorders will be comprehensively searched without restrictions on language or publication date. Additionally, unpublished documents were manually searched. Exclusion of review literature; literature on non-clinical study types; retrospective trials; prescription statistics; literature in which adverse reaction outcomes were not given; trials in which the Western medicine group and the combined Western and Chinese/Western medicine group combined other treatments in addition to standard care, Western medicine treatment and combined Western and Chinese medicine treatment; trials in which the combined Western and Chinese medicine group had patients taking both tonics and proprietary Chinese medicine; studies in which only 1 symptom of neocrown pneumonia was treated; taking medicine before and after self-control trials; trials in which the literature indicated that the grouping was non-randomized (trials with pseudo-randomized grouping were not included in the exclusion criteria but were included in the quality analysis of the literature).

### 2.3. Types of participants

Subjects with documented COVID-19 with olfactory disorders lasting 2 weeks or longer. There were no restrictions on sex, education, race, or disease stage, aged between 18 and 65 years. Patients with a history of olfactory disorders prior to COVID-19 infection were excluded.

### 2.4. Types of interventions and comparisons

COVID-19 treatment was used as routine treatment. Intervention: Chinese and Western medicine combined with soup or Chinese medicine combined with Western medicine for the treatment of neoconjunctivitis in the Chinese and Western medicine group; Control: Western medicine alone with the same Western medicine as in the Chinese and Western medicine group for the treatment of neoconjunctivitis; The outcome index was the occurrence of adverse reactions.

### 2.5. Types of outcomes

The following information was collected from each study: publication year, country, ethnicity, specimen source, sex and age, case and control numbers, primary outcomes, and safety outcomes. All database searches, literature screening, and information extraction were conducted by 2 researchers according to predetermined rules and criteria, and in case of disagreement, a joint decision was made after discussion with a third person.

#### 2.5.1. Primary outcomes.

Clinical variables will be set as the primary outcomes, such as olfactory disorders frequency, duration of olfactory disorders, duration of use of medicines, and quality of life.

#### 2.5.2. Safety outcomes.

The incidence of adverse events.

### 2.6. Search strategy

According to the PICO principles of the Cochrane Manual for the Systematic Evaluation of Interventions (hereinafter referred to as “the Cochrane Manual”) [P: participants; I: interventions; C: comparisons O:outcomes] and the treatment protocols recommended in the “New Coronavirus Pneumonia Protocols (First to Eighth Editions),” combined with the clinical practice for the use of drugs for new coronavirus pneumonia, to set the search terms.

The Cochrane Central of Controlled Trials (CENTRAL), PubMed, EMBASE, Cochrane Library, Web of Science, Chinese Biomedical Databases, China National Knowledge Infrastructure, Wanfang Database, and VIP Database. Using different databases, we combined keywords and free words for a comprehensive search. The complete PubMed search strategy is shown in Table 1.

**Table 1:** Search strategy for PubMed.

Number	Search items
#1	“Chinese medicine” [Title/Abstract] OR “Chinese medicine” [Title/Abstract] OR “Chinese and Western medicine” [Title/Abstract] OR “Chinese and Western medicine” [Title/Abstract] OR “soup” [Title/Abstract] OR “Chinese patent medicine” [Title/Abstract] OR “clear lung detox soup” [Title/Abstract] OR “patchouli” [Title/Abstract] OR “golden flower clearing sensation” [Title/Abstract] OR “even flower clearing plague” [Title/Abstract] OR “dredge wind and detoxify” [Title/Abstract] OR “prevent wind and pass sage” [Title/Abstract] OR “add flavor mulberry chrysanthemum drink” [Title/Abstract] OR “transform dampness and defeat toxin formula”[Title/Abstract].
#2	“covid 19”[Title/Abstract] OR “2019-nCoV”[Title/Abstract] OR “coronavirus disease 19” [Title/Abstract] OR “coronavirus disease 2019”[Title/Abstract] OR “disease 2019 coronavirus”[Title/Abstract] OR “sars coronavirus 2 infection”[Title/Abstract] OR “SARS-CoV- 2”[Title/Abstract].
#3	“Olfactory impairment” [title/abstract] or “Olfactory disorder” [title/abstract] or “Olfactory abnormality” [title/abstract] or “Olfactory malfunction” [title/abstract].
#4	“Randomized controlled trial” [Title/Abstract] OR “Controlled clinical trial “ [Title/Abstract] OR “clinical tria lrandomized“ [Title/Abstract]
#5	“Delta” [Title/Abstract] OR “Delta strains” [Title/Abstract] OR “Delta mutant strains” [Title/Abstract].
#6	#1 and #2 and #3 and #4 and #5.

The search strategy would be modified as required for other databases. According to different databases, keywords can be combined with free words for comprehensive search.

### 2.7. Data collection and analysis

#### 2.7.1. Selection of studies.

**Figure 1.**
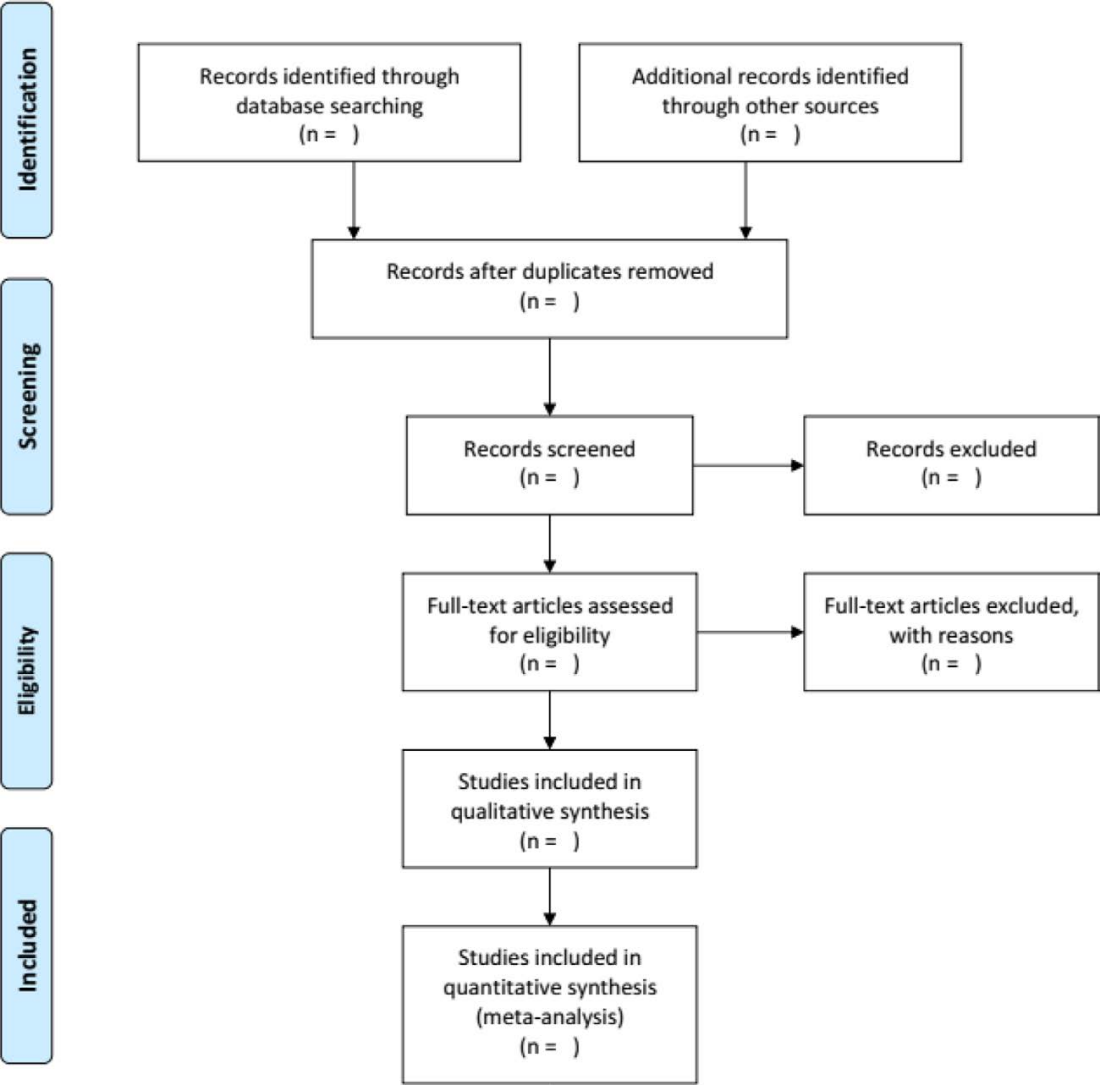
The PRISMA flow diagram. PRISMA = preferred reporting items for systematic reviews and meta-analysis.

The search strategy and study selection were independently performed by 2 researchers (XHL and QG) in a database search, and the final research choices were agreed upon. Two researchers independently evaluated the same article to determine their eligibility for inclusion and resolved differences through consensus. The further unresolved discrepancy was managed by a third reviewer (YFW). The selection process was summarized using a PRISMA flow diagram. The details of the selection procedure for the studies are shown in the PRISMA flow chart (Fig. 1).

#### 2.7.2. Data extraction and management.

Two researchers (XHL and QG) from the database for data extraction and data management, including the basic information of the included studies, baseline characteristics of the subjects, intervention and control measures, key elements of bias risk assessment, and outcome indicators. If there is any disagreement between the 2 researchers during the data extraction process, the panel jointly arbitrates and makes a decision.

#### 2.7.3. Dealing with missing data.

If the information is missing or unclear, we will try to contact the corresponding author for more detailed information. If it fails, we analyze it based on the available data.

#### 2.7.4. Assessment of risk of bias.

The risk of bias was assessed using the risk of bias assessment tool recommended in the Cochrane Handbook, and the risk of bias bar graphs and risk of bias summary graphs were created using the software Review Manager 5.4. The assessment consisted of 7 main entries: random sequence generation; Allocation concealment; Blinding of participants and personnel; Blinding of outcome assessment. Incomplete outcome data; Selective reporting; Other bias.

Two researchers will determine bias based on the following items: random sequence generation, allocation concealment, blinding of the participants and personnel, blinding of the outcome assessments, incomplete outcome data, selective reporting, and other sources of bias. Studies will be evaluated as “low-risk, high-risk,” or “clear risk.” Inconsistencies were resolved by discussion with other reviewers (YFW).

#### 2.7.5. Assessment of quality of evidence.

All studies used the Newcastle-Ottawa Scale assessment scale^[10]^ for evaluation. The evidence quality will be evaluated by 2 viewers (YFW and QG) independently with the grading of recommendations assessment, development, and evaluation. The scale includes 9 items, covering 3 dimensions. The study was awarded 1 point for each item. The Newcastle-Ottawa scale scores range from 0 to 9, with higher scores indicating better quality. In this study, a score of ≥ 6 was considered to be of better quality.

#### 2.7.6. Measures of treatment effect.

Review Manager software (RevMan 5.3) was used to conduct this meta-analysis. For continuous results, the data were expressed as the mean difference (MD) or standard mean difference (SMD) with a 95% confidence interval. When dichotomous data were available, a hazard ratio with 95% confidence interval was used. When binary data exist, the RR format is changed for the analysis.

#### 2.7.7. Heterogeneity evaluation.

Clinical heterogeneity and statistical heterogeneity between the studies were assessed. Clinical heterogeneity was judged according to the similarity of research objects, intervention measures, control, and outcome indicators, and statistical heterogeneity was evaluated by *I*^2^. If *I*^2^ ≤ 50% and *P* > .1, statistical homogeneity was considered to be good. A fixed-effects model was used for merging. If *I*^2^ > 50% or *P* ≤ .1, the statistical heterogeneity was large, and the source of heterogeneity was further analyzed. After obvious clinical heterogeneity was excluded, a random-effects model was used for meta-analysis. When there is obvious clinical heterogeneity, it should be treated by subgroup analysis, sensitivity analysis, or only descriptive analysis.

#### 2.7.8. Assessment of reporting bias.

When outcomes included more than 10 studies, we used Stata 14.0 to assess the reporting bias using a funnel plot and Egger’s test.^[11]^

#### 2.7.9. Data synthesis.

We took advantage of Review Manager software (RevMan 5.3) for data analysis and synthesis. If there was no statistical heterogeneity between the results, a fixed-effects model was used. If there was statistical heterogeneity between the results, a random-effects model was used. If there was significant clinical heterogeneity, subgroup or sensitivity analysis was performed.

#### 2.7.10. Subgroup analysis.

If feasible, we will conduct a subgroup analysis based on different interventions, controls, treatment duration, and outcome indicators.

#### 2.7.11. Sensitivity analysis.

We carried out a sensitivity analysis to investigate the robustness of the conclusions. The principal decision nodes include the method quality, sample size, and impact of missing data. Therefore, the impact of low-quality research on overall results was assessed.

#### 2.7.12. Ethics and dissemination.

Since this study did not involve patient privacy, ethical approval was not required. Our research results will be shared and presented through conference reports and peer-reviewed journals.

## 3. Discussion

This study aimed to evaluate the efficacy and safety of Chinese herbal tonics for the treatment of olfactory disorders in COVID-19. olfactory disorders belong to the category of “epistaxis” in traditional Chinese medicine, and as an external treatment method of traditional Chinese medicine, Chinese herbal tonics has the characteristics of simple and simple verification. It can not only prevent the occurrence of diseases but can also be used as an auxiliary treatment after the occurrence of diseases.

This study provides a new choice for a variety of treatment options for olfactory disorders in COVID-19. We hope that this review will provide more convincing evidence for clinicians to treat these conditions and help them make appropriate decisions.

## Author contributions

All the authors had access to the data and played a role in writing the manuscript. All authors have read and approved the final manuscript.

**Data curation:** Xuhao Li.

**Formal analysis:** Qing Gao.

**Methodology:** Xuhao Li.

**Project administration:** Qing Gao.

**Resources:** Qing Gao.

**Software:** Xuhao Li and Yifeng Wang.

**Visualization:** Yifeng Wang.

**Writing – original draft:** Xuhao Li and Qing Gao.

**Writing – review & editing:** Xuhao Li.
